# Effect of sex, pubertal stage, body mass index, oral contraceptive use, and C-reactive protein on vitamin D binding protein reference values

**DOI:** 10.3389/fendo.2025.1470513

**Published:** 2025-02-18

**Authors:** Philipp von Heimburg, Ronny Baber, Anja Willenberg, Philip Wölfle, Jürgen Kratzsch, Wieland Kiess, Mandy Vogel

**Affiliations:** ^1^ LIFE Child - Leipzig Research Center for Civilization Diseases, Leipzig University, Leipzig, Germany; ^2^ Institute for Laboratory Medicine, Clinical Chemistry and Molecular Diagnostics (ILM), Leipzig University, Leipzig, Germany; ^3^ German Center for Child and Adolescent Health (DZKJ), partner site Leipzig/Dresden, Leipzig, Germany; ^4^ Department of Women and Child Health, Hospital for Children and Adolescents and Center for Pediatric Research (CPL), Leipzig University, Leipzig, Germany

**Keywords:** vitamin D binding protein, DBP, reference values, BMI, obesity, contraceptive drugs, pubertal stage

## Abstract

**Objective:**

Vitamin D binding protein (DBP) regulates the transport and availability of vitamin D. We aimed to establish age- and sex-specific reference ranges for serum concentrations of DBP in healthy infants, children, and adolescents. In addition, we investigated DBP’s associations with age, sex, puberty, body mass index (BMI), and oral contraceptive use.

**Design and methods:**

2,503 serum samples from children and adolescents aged 3 months to 17 years from the LIFE Child cohort were analyzed to study DBP levels in this population (49.3% female subjects, 50.7% male subjects). Age- and sex-dependent reference percentiles were established using generalized additive models. We used linear mixed effects models to assess DBP’s associations with age, sex, pubertal status, the BMI standard deviation score (SDS), and oral contraceptives. To investigate associations between DBP and vitamin D metabolites, we applied univariate regression analysis. We used hierarchical regression models and linear mixed effects models to assess DBP’s associations with bone parameters, hormones, and inflammatory markers.

**Results:**

Mean DBP values differed between males (347 mg/l) and females (366 mg/l) (p < 0.001). Age had no significant association with DBP levels. In both males and females, DBP levels remained relatively stable from infancy through late adolescence. Children and adolescents with obesity had lower mean DBP levels compared with normal-weight subjects (ß = -14.28, p < 0.001). The BMI-SDS was inversely associated with DBP levels in males (ß = -5.7, p < 0.001). Female subjects using oral contraceptives had higher levels of DBP (ß = 141.38, p < 0.001). DBP was positively associated with the vitamin D metabolites: 25(OH)D_3_ (females: ß = 0.8, p < 0.001; males: ß = 1.2, p < 0.001) and 1,25(OH)_2_-D_3_ (females: ß = 0.3, p < 0.001; males: ß = 0.4, p < 0.001). An inverse association between osteocalcin and DBP (females: ß = -0.1, p < 0.022; males: ß = -0.1, p = 0.027) was found. CRP levels were also positively associated with DBP levels (females: ß = 2.8, p = 0.001; males: ß = 5.1, p < 0.001).

**Conclusion:**

We established age- and sex-specific reference ranges for the serum concentration of DBP. We suggest that BMI, pubertal stages, oral contraceptive use, and inflammation markers need to be considered when interpreting DBP as a stabilizer and regulator of vitamin D metabolism and vitamin D status in children and adolescents.

**Clinical trial registration:**

ClinicalTrial.gov, identifier NCT02550236.

## 1 Introduction

Besides albumin, vitamin D binding protein (DBP), also known as GC-globulin, binds to all vitamin D metabolites, creating a large pool of circulating 25-hydroxyvitamin D (25(OH)D_3_) and 1,25-dihydroxyvitamin D (1,25(OH)_2_-D_3_), which helps balance bone metabolism and prevent rapid vitamin D deficiency. Additionally, DBP is known as an acute-phase reactant and plays a multifaceted role during inflammation (1–3). Despite the physiologically important properties that have been attributed to DBP, there is a limited understanding of the influence of the plasma concentration of DBP in healthy individuals (4). The current understanding is that DBP, which is produced in the liver, is not regulated by vitamin D itself (2). Production increases after exposure to estrogen, glucocorticoids, and inflammatory cytokines, such as interleukin-6 (5). Oral contraceptives are also known to increase the synthesis of serum globulins. The effect depends on the type of estrogen and the dosage (6). On the other hand, preparations containing progesterone with an androgenic effect may attenuate the effect of estrogen (6). However, the mechanisms behind these effects are not yet understood (7, 8). Patients with, for example, kidney or liver disease, malnutrition, or type 1 diabetes were found to have lower DBP levels (9). The influence of excess body fat on DBP serum levels is still unknown because recent studies have reported positive (10, 11) or negative (4) associations with different measures, e.g., BMI. No significant association has been found between age and DBP concentration in adults so far (12). While extensively studied in adults, research on DBP in children and adolescents is limited. Therefore, we aimed to describe DBP levels across childhood and adolescence to thereby establish age- and sex-dependent reference intervals based on DBP serum levels from more than 1,800 healthy children and adolescents from the LIFE Child cohort. Furthermore, we assessed how DBP levels are associated with BMI and oral contraceptive use. In addition, we investigated DBP’s relationships with bone metabolism (phosphate, osteocalcin, alkaline phosphatase, calcium, parathormone), sex hormones (estradiol, progesterone, testosterone), protein synthesis in the liver (sex hormone-binding globulin, albumin), and an inflammation marker (C-reactive protein).

## 2 Material and methods

### 2.1 Ethical considerations

The LIFE Child study is registered with ClinicalTrial.gov (NCT02550236) and was approved by the Ethical Committee of the Medical Faculty of the University of Leipzig (Reg. No. 264-10-19042010). The procedures were performed in accordance with the ethical standards of the Declaration of Helsinki. The serum samples were collected after receiving informed written consent from the parents and, for subjects age 12 and up, the subjects themselves. Data were collected between May 2011 and December 2014. Detailed information regarding the recruitment and the examinations is given in Poulain et al. and Quante et al. (13, 14).

### 2.2 Study population and design

**Figure 1:**
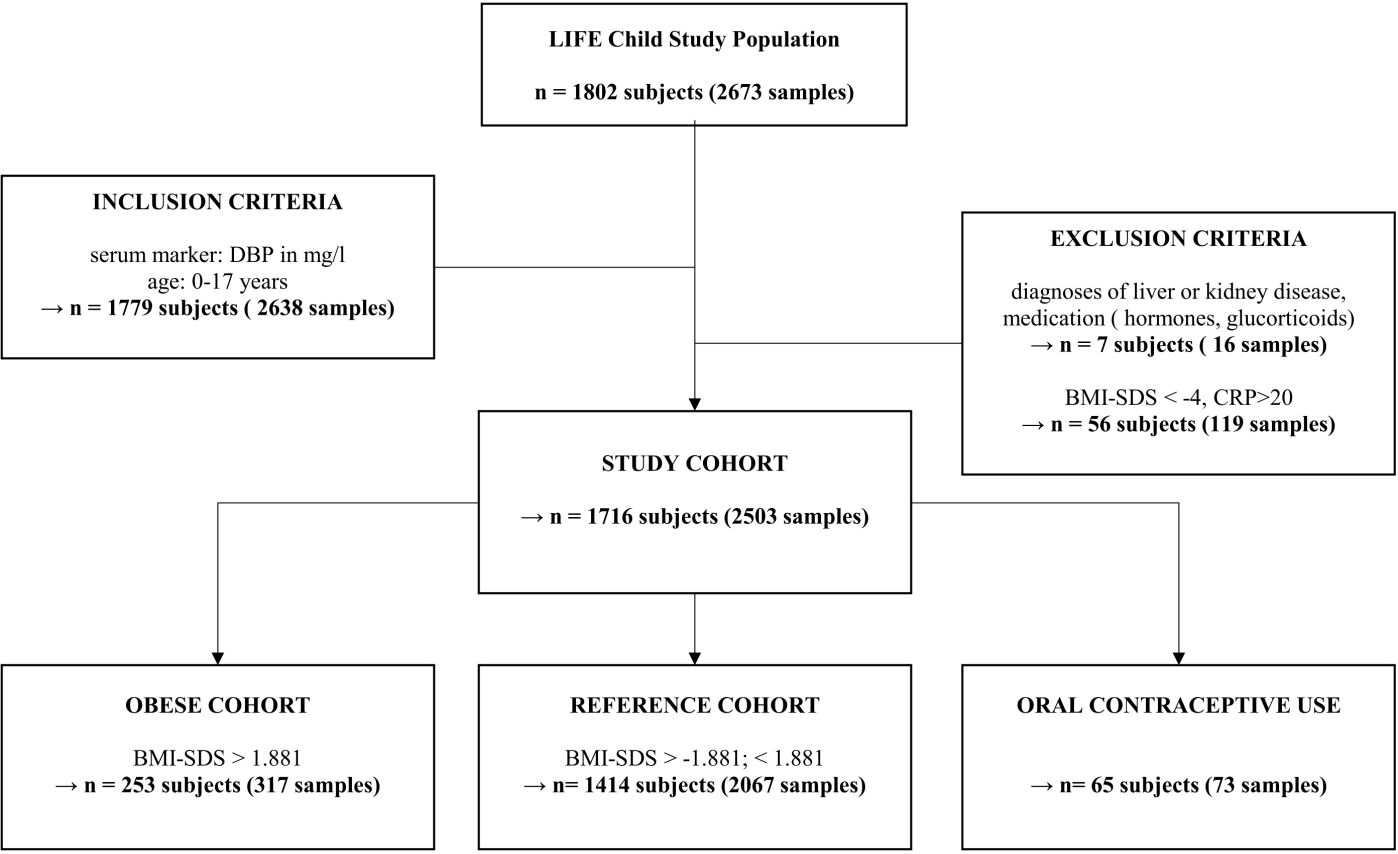
Inclusion and exclusion based on multiple criteria for our study cohort of children and adolescents. “n” represents the number of subjects or the number of samples across multiple study visits. Due to multiple visits, a subject may, for example, be a member of the reference cohort at their first visit but part of the obese cohort at a later visit. Oral contraceptive use subgroup includes all females with sexual hormone binding globulin levels ≥ 200 ng/ml and age >13 years (46 samples from 42 subjects) and those who reported taking oral contraception (27 samples from 23 subjects). DBP, Vitamin D binding protein; CRP, C-reactive protein; BMI, body mass index.

The LIFE Child study is a longitudinal, prospective, and population-based cohort study and is part of the Leipzig Research Center for Civilization Diseases (LIFE) in the city of Leipzig (Saxony, Germany). Participants’ ages range from 3 months to 20 years. Most of the study participants originate from Leipzig or its immediate surroundings. They undergo an age-specific study program with yearly follow-up visits that include various medical, psychological, and sociodemographic assessments, as well as the collection of biological samples. The study was designed to examine the influence of genetic, metabolic, and environmental factors on children’s growth, development, and health. A total of 2,673 serum samples from 1,802 subjects were available (**Figure 1**). Medication, diseases, or physiological circumstances that may affect DBP and vitamin D metabolism, such as concomitant kidney diseases, liver diseases, severely underweight (BMI-SDS < -4), glucocorticoid therapy, or pregnancy were excluded (2, 5, 15–17). In line with recent recommendations, we excluded subjects with C-reactive protein (CRP) > 20mg/l (18). Subjects older than 17 were excluded because this subgroup had such a small sample size. The final data set comprised 2,503 samples from 1,716 subjects between 0.25 and 17 years of age. Out of this study population three sub cohorts for additional analysis were established. For the reference cohort, children and adolescents using oral contraceptives or with BMI-SDS < -1.881 or > +1.881 were excluded. We did not exclude subjects with vitamin D supplementation because it is recommended for all infants in Germany and does not seem to affect DBP serum levels (19). 2,067 samples from 1,414 subjects were included to establish reference values. To assess the association between BMI-SDS and DBP, we established a sub cohort including only the individuals with BMI-SDS > 1.881 (317 samples from 253 individuals). In addition, to compare the DBP levels of women who reported (27 samples from 23 subjects) or were thought to be taking oral contraceptives with the corresponding population of women who were not taking oral contraceptives in total 73 samples from 65 subjects were included. This sub cohort contains, in line with Hörenz et al., all females with sexual hormone binding globulin (SHBG) levels ≥ 200 ng/ml and age >13 years (46 samples from 42 subjects) who did not report oral contraceptive use but were assumed to be taking oral contraceptives as well (20).

### 2.3 Anthropometric measures and pubertal status

Height, weight, and pubertal status were assessed by trained study assistants. BMI was calculated and transformed into age- and sex-adjusted standard deviation scores (SDS) in accordance with the S3 guidelines of the German Obesity Society and the German Society of Pediatrics and Adolescent Medicine (21). Accordingly, obesity was defined as having a BMI-SDS > 1.881, overweight as a BMI-SDS of 1.28 to 1.881, normal weight as a BMI-SDS of -1.28 to < 1.28, underweight as a BMI-SDS of < -1.28 to -1.881, and extreme underweight as a BMI-SDS < -1.881. Pubertal status was measured as Tanner stages (22, 23).

### 2.4 Laboratory measurements

Venous blood was drawn from the study subjects by trained personnel in the morning (with 90% collected between 07:30 and 10:00, 96% by 11:00, 99% by 12:00, and 100% by 16:00) (24). Parts of the samples were processed by trained staff from the Leipzig Medical Biobank and sent to the Institute of Laboratory Medicine, Leipzig, on the same day for analysis. The rest were aliquoted, frozen, and stored at -80°C or -150°C in the LIFE-Biobank for additional analytical procedures (14). To determine the DBP values, an automated immune turbidimetric assay was performed as a DAKO-derived free application for the Gc-globulin determination in serum using the c-module of the Cobas^©^ 8000 system from the Roche Diagnostics GmbH (Germany, 25, 26). The intra- and interassay coefficients of variation for a pool serum with a level between 266 and 271 mg/l were below 1.8% and below 2.1%, respectively. Albumin, SHBG, and CRP were analyzed with a Cobas^©^ 6000/8000 Clinical Chemistry Analyzer with test kits from Roche Diagnostics GmbH in accordance with the clinical chemistry routine. Cobas^©^ 601 and 801 from Roche were used to measure bone metabolism parameters (osteocalcin) and calcium homeostasis (parathormone, 25(OH)D_3_ total). 1,25(OH)_2_-D_3_ was measured with enzyme immunoassays in the IDS-iSYS Multi-Discipline Automated System. Steroid hormones were analyzed with liquid chromatography tandem mass spectrometry, which was described in detail by Gaudl et al. (27).

### 2.5 Statistical analysis and calculation of references

Descriptive statistics are presented as counts (percentages) for categorical variables and means for continuous variables (**Table 1**). We considered p < 0.05 to be statistically significant. All statistical analyses were computed in R (version 4.2.1) (28). Generalized additive models for location scale and shape were used to estimate age- and sex-adjusted reference intervals for DBP (29), assuming a Box-Cox-Cole-and-Green distribution. To avoid multicollinearity caused by correlated laboratory measures, laboratory measures were clustered using hierarchical clustering as a basis for the subsequent regression analyses. The number of clusters was determined by visual inspection (**Supplementary Figure S1**). Only one representative per cluster was included in the multivariate models. Eight parameters were identified as representatives of their clusters. The final models were stratified by sex, as sex hormones were included as predictor variables. Non-significant variables were removed from the model in a stepwise fashion (backward deletion). Only parameters that were of particular interest for our research question were retained despite non-significance. Univariate regression analysis was applied to investigate the relationships between DBP and its metabolites. Differences in DBP serum levels between males and females, as well as between the reference and obesity groups, were estimated with linear mixed-effects models. Beta coefficients were used as measures of effect size to indicate relationship strength and direction. All models listed so far were adjusted for multiple measurements per subject by adding the subject as a random intercept. We computed t-tests to compare the mean values between Tanner stages.

**Table 1:** Data analysis and description of study cohort.

	overall	female	miss.	mean DBP (mg/l)	male	miss.	mean DBP (mg/l)
n	2,503^1^	1,234^1^		366	1,269^1^		347*** ^2^
		(49.3%)			(50.7%)		
age (years)	10.9 (3.5)	11.2 (3.6)			10.7 (3.4)		
BMI-SDS							
extreme underweight	52 (2.1%)	23 (1.9%)		372	29 (2.3%)		348
underweight	141 (5.6%)	64 (5.2%)		375	77 (6.1%)		358
normal	1,816 (73%)	889 (72%)		369	927 (73%)		351
overweight	177 (7.1%)	100 (8.1%)		354	77 (6.1%)		337
obese	317 (13%)	158 (13%)		352	159 (13%)		324
Tanner stages							
1	941 (47%)	427 (37%)		355	514 (59%)		350
2	357 (18%)	184 (16%)		355	173 (20%)		356
3	203 (10%)	147 (13%)		357	56 (6.5%)		337* ^3^
4	237 (12%)	163 (14%		362	74 (8.5%)		331** ^3^
5	268 (13%)	218 (19%)		357	50 (5.8%)		332** ^3^
miss.	497	95			402		
albumin (g/l)	47.75 (2.73)	47.64 (2.72)	3		47.87 (2.74)	5	
25(OH)D (ng/ml)	22 (10)	22 (10)	1		23 (10)	4	
1,25(OH)2-D3 (pg/ml)	59 (20)	62 (20)	110		57 (19)	117	
SHBG (nmol/l)	84 (51)	87 (56)	335		81 (45)	384	
estradiol (pmol/l)	85 (140)	125 (187)	782		43 (16)	837	
testosteron (nmol/l)	2.9 (5.8)	0.6 (1.2)	555		5.0 (7.4)	565	
osteocalcin (ng/ml)	97 (41)	88 (39)	334		106 (41)	369	
alkaline phosphatase (µkat/l)	3.72 (1.60)	3.31 (1.41)	7		4.13 (1.68)	10	
parathormon (pmol/l)	3.50 (1.27)	3.57 (1.25)	324		3.42 (1.30)	351	
calcium (mmol/l)	2.49 (0.09)	2.49 (0.09)	8		2.49 (0.09)	8	
phosphate (mmol/l)	1.48 (0.18)	1.45 (0.18)	7		1.50 (0.17)	11	
CRP (mg/l)	1.08 (1.91)	1.17 (1.96)			0.99 (1.85)		
oral contraceptive use		73 (2.9%)		508*** ^4^			

Due to multiple visits, a subject may, for example, be a member of the reference cohort at their first visit but part of the obese cohort at a later visit. BMI-SDS groups: extreme underweight: BMI-SDS < -1.881; underweight: BMI-SDS -1.881 to < -1.28; normal weight: BMI-SDS -1.28 to < 1.28; overweight: BMI-SDS 1.28 to 1.88; obese: BMI-SDS > 1.88. BMI-SDS = body mass index standard deviation score; p = significance codes: < 0.001 “***”, < 0.01 “**”, < 0.05 “*”; CRP, C-reactive protein; DBP, Vitamin D binding protein; SHBG, sex hormone binding protein; miss, missing; ^1^mean (SD), ^2^t-test (female vs male), ^3^linear mixed model: reference level = Tanner stage 1 (male), ^4^t-test (female BMI-age matched control Tanner stage 4 and 5), n (%).

“n” represents the number of samples across multiple study visits.

## 3 Results

### 3.1 Characteristics of the study cohort

**Table 1** presents descriptive statistics for the sample. 1,716 subjects with 2,503 observations (49.3% females, 50.7% males) were included. The mean age (all measurements) was 10.9 ± 3.5 years (range: 0.94 – 17.98 years). In total, there were 73 samples from females taking oral contraceptives. 73% of all samples came from normal-weight subjects, 7.7% from underweight and extreme underweight subjects, 7.1% from overweight subjects, and 13% from subjects with obesity.

### 3.2 DBP percentiles – age and sex differences

**Figure 2:**
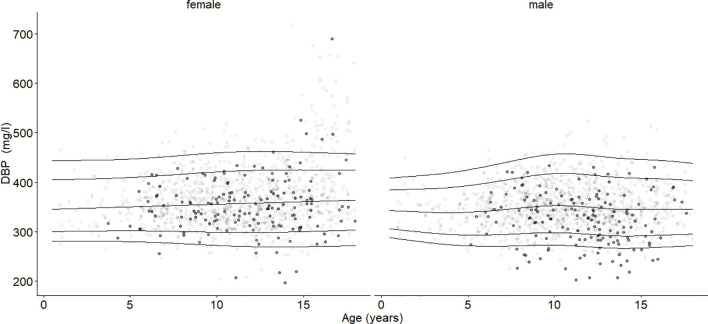
Age- and sex-adjusted reference percentiles (2.5th, 10th, 50th, 90th, and 97.5th) for DBP (mg/l) for the reference cohort: Subjects in the study cohort (grey dots) were compared with subjects in the obesity cohort (black dots, BMI-SDS ≥ 1.881). Age had no significant association. DBP values in the group with obesity were significantly lower than DBP in the reference group, age-adjusted and independent of sex (ß = -14.28, p < 0.001).

**Figure 3:**
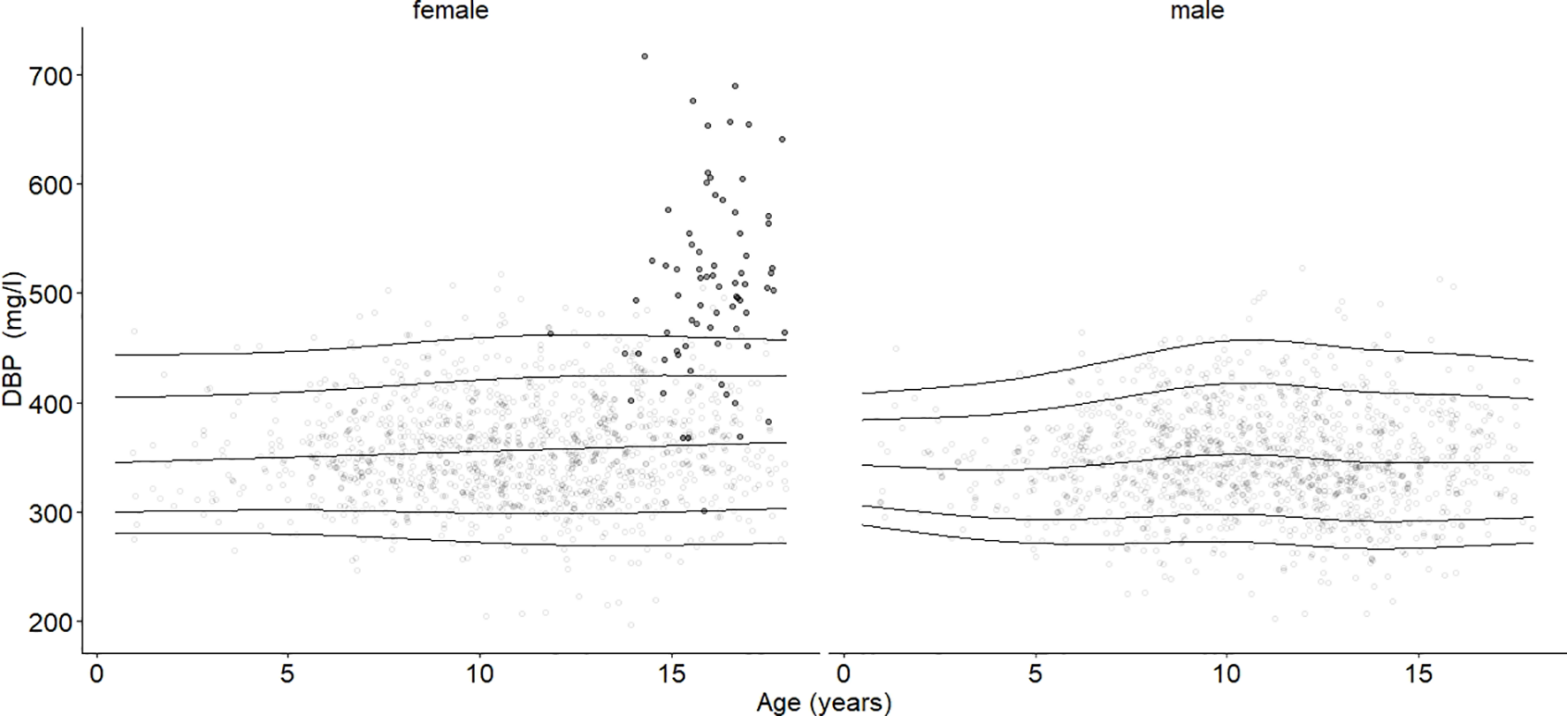
Age- and sex-adjusted 2.5th, 10th, 50th (median), 90th, and 97.5th percentiles for DBP (mg/l) for the reference cohort, subjects in the study cohort (grey dots), and subjects with oral contraceptive use (black dots). Subjects using oral contraceptives had increased DBP blood levels (p < 0.001).

The 2.5th, 5th, 10th, 25th, 50th, 75th, 90th, 95th, and 97.5th percentiles for the reference cohort stratified by sex at different ages are shown in **Table 2**. The DBP smoothed percentile curves (**Figures 2**, **3**) showed a similar pattern in males and females. From infancy until late adolescence, DBP levels remained relatively stable. In the higher percentiles (P90-P97.5), the values were predominantly higher in females than males. The gap between females’ and males’ measurements was particularly large between the ages of 12 and17.98. The lower percentiles (P2.5-P10) did not differ markedly between males and females. For DBP, the 50th percentile remained relatively stable for males from 8 to 17.98 years of age, while it increased slightly but continuously in females. The increase was small (ß = 1.3 mg/l/year) but significant (p = 0.006) for girls. There was no similar trend for boys.

**Table 2:** Percentiles derived from generalized additive models for location, scale, and shape for males and females in the reference cohort.

	percentiles of DBP (mg/l)											
Sex	age	n	P2.5	P5	P10	P50	P90	P95	P97.5	mu	sigma	nu
male	2	11	281	290	300	340	386	400	412	340.16	0.10	-0.02
	4	26	273	283	294	338	389	405	419	338.24	0.11	-0.02
	6	66	270	281	293	341	398	415	431	341.34	0.12	-0.02
	8	142	271	282	296	348	409	428	446	347.52	0.13	-0.02
	10	228	272	284	298	352	417	438	457	352.45	0.13	-0.02
	12	206	269	281	294	349	415	436	454	349.43	0.13	-0.02
	14	213	266	277	291	345	409	429	448	344.92	0.13	-0.02
	16	135	268	279	292	345	407	426	444	344.89	0.13	-0.02
	18	54	272	282	295	345	403	421	438	344.67	0.12	-0.02
females	2	10	280	289	301	347	406	426	444	346.71	0.12	-0.65
	4	25	280	290	301	349	408	427	445	348.84	0.12	-0.45
	6	54	279	289	301	351	411	431	449	350.96	0.12	-0.25
	8	147	275	287	300	353	416	436	454	353.08	0.13	-0.06
	10	176	272	284	299	355	421	441	459	355.19	0.13	0.14
	12	188	270	283	298	357	424	444	462	357.30	0.14	0.34
	14	196	269	283	299	359	425	444	462	359.40	0.14	0.54
	16	120	270	284	301	362	425	443	459	361.50	0.13	0.74
	18	70	271	286	303	364	425	442	457	363.60	0.13	0.94

DBP, Vitamin D binding protein; n, amount of samples.

### 3.3 DBP’s associations with age, sex, and pubertal status

**Figure 4:**
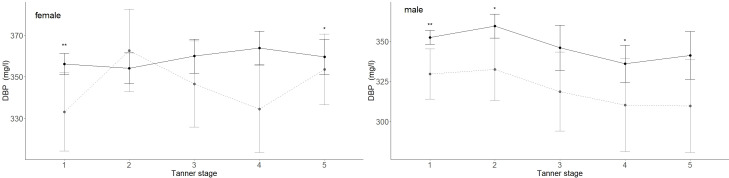
DBP (mg/l) values for children and adolescents from the reference (black lined) and obese cohorts (grey dashed line) are shown by Tanner stages. Females taking contraceptive drugs were excluded from this analysis. The 95% confidence intervals are represented by vertical lines. The numbers are indicated in **Supplementary Table S2**. Mean DBP levels in obese males were significantly lower in Tanner stages 1, 2, and 4 and significantly lower in females in Tanner stages 1 and 5. p-values: p = significance codes: < 0.01 “**”, < 0.05 “*”.

The mean DBP values differed between males (347 mg/l) and females (366 mg/l) (p < 0.001). Age had no significant association with the median DBP serum level (**Figure 2**). DBP peaked at Tanner stage 4 in females (362 mg/l) and at Tanner stage 2 in males (356 mg/l) (**Figure 4**). DBP levels were significantly lower in males than females during Tanner stages 3, 4, and 5 (**Supplementary Table S1**). As presented in **Table 1**, DBP levels were significantly lower in males during Tanner stages 3, 4, and 5 compared with pre-pubertal males (Tanner stage 1). In females, on the other hand, no significant relationship was observed between Tanner stage and DBP values.

### 3.4 Association between BMI and DBP

**Figure 5:**
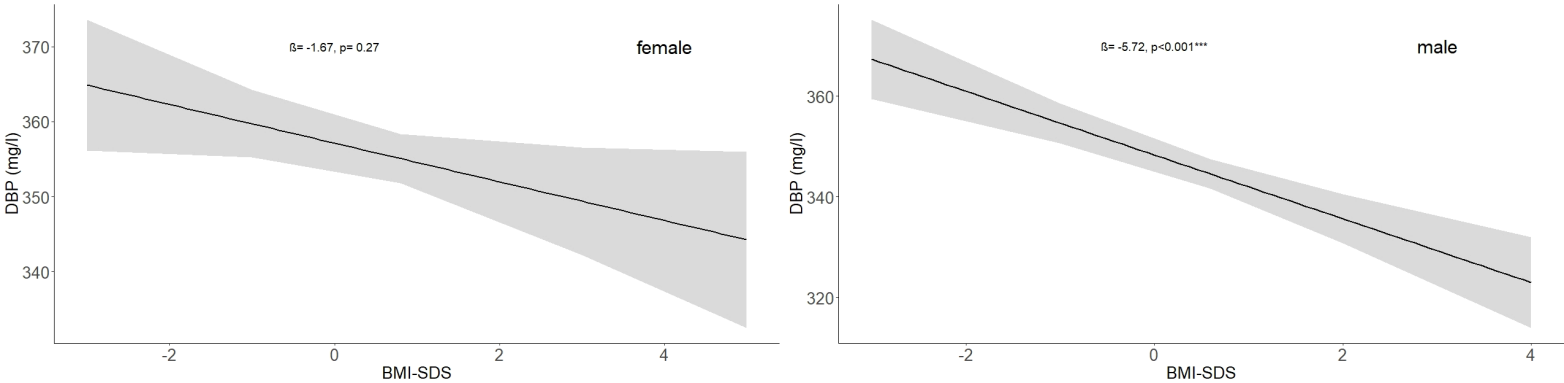
Association between BMI-SDS and DBP (mg/l) values in the study cohort for males and females with no subjects taking oral contraceptives (females: ß = -1.67, p = 0.27; males: ß = -5.72, p < 0.001). The numbers of subjects are indicated in **Table 1**.

In the study cohort (1716 subjects and 2503 samples) DBP was inversely associated with BMI-SDS, but the association was statistically significant only in males (p < 0.001). While an increase in BMI-SDS of +1 showed a decrease of 5.72 mg/l in the DBP serum concentration in males, it decreased by only 1.67 mg/l in females (**Table 3**, **Figure 5**). DBP values in the group with obesity were significantly lower than DBP in the reference group, age-adjusted and independent of sex (ß = -14.28, p < 0.001). These findings are illustrated in **Figure 2**. Males with obesity (159 samples from 130 subjects) had significantly lower DBP levels than their reference weight peers (1081 samples from 742 subjects) in Tanner stages 1, 2, and 4. Similarly, females with obesity (158 samples from 123 subjects) had significantly lower levels in Tanner stages 1 and 5 than their reference group (986 samples from 672 subjects) (**Figure 4**; **Supplementary Table S2**).

**Table 3:** Multivariate associations between DBP and various variables in the study cohort, excluding subjects with oral contraceptive use.

	DBP (mg/l)					
	females			males		
	estimate	b	p	estimate	b	p
BMI-SDS	-1.67	-0.04	0.265	-5.72	-0.14	< 0.001***
osteocalcin	-0.1	-0.08	0.022*	-0.08	-0.07	0.027*
albumin	4.01	0.23	< 0.001***	3.15	0.19	< 0.001***
CRP	2.78	0.1	0.001**	5.09	0.18	< 0.001***
SHBG				0.11	0.11	0.005**

For all predictors, estimate = ß, the standardized effect size beta (b) is given, including the respective p-values. p = significance codes: < 0.001 “***”, < 0.01 “**”, < 0.05 “*”, BMI-SDS, Body mass index standard deviation score; DBP, Vitamin D binding protein; CRP, C-reactive protein; SHBG, sexual hormone binding protein.

**Table 3A:** Univariate associations between DBP and its metabolites in the study cohort excluding subjects with oral contraceptive use.

	DBP (mg/l)					
	females			males		
	estimate	b	p	estimate	b	p
25(OH)D	0.81	0.22	< 0.001***	1.20	0.34	< 0.001***
1,25(OH)_2_-D_3_	0.30	0.21	< 0.001***	0.41	0.27	< 0.001***

For all predictors, estimate = ß, the standardized effect size beta (b) is given, including the respective p-values. p = significance codes: < 0.001 “***”, DBP, Vitamin D binding protein; 25(OH) D, 25-hydroxyvitamin D; 1,25(OH)_2_-D_3_, 1,25-dihydroxyvitamin D.

### 3.5 Oral contraceptive use and DBP

DBP values from subjects taking oral contraceptives are highlighted in **Figure 3** (females). The mean DBP value (508 mg/l) was significantly higher (estimate = 141.38, b=0.69, p < 0.001) compared with age-BMI matched subjects in Tanner stage 4 and 5, independent of the oral contraceptive formulations. Furthermore, we found that estradiol serum levels in female subjects who were not taking oral contraceptives were not significantly associated with DBP levels.

### 3.6 DBP’s associations with vitamin D metabolites, bone metabolism, acute phase reactants, and protein synthesis

In both sexes, significant positive associations were found between DBP and the vitamin D metabolites: 25(OH)D_3_ (females: ß = 0.8, p < 0.001; males: ß=1.2, p<0.001) and 1,25(OH)_2_-D_3_ (females: ß = 0.3, p < 0.001; males: ß = 0.4, p < 0.001) (**Table 3A**). The analysis presented in **Table 3** showed a significant inverse association between osteocalcin and DBP (females: ß = -0.1, p < 0.022; males: ß = -0.1, p = 0.027). A significant relationship was found between albumin and DBP (females: ß = 4.0, p < 0.001; males: ß = 3.2, p < 0.001). In males but not in females, there was also a significantly positive association between SHBG and DBP (ß = 0.1, p = 0.005). A statistically significant positive relationship between CRP levels (< 20mg/l) and the serum concentration of DBP (females: ß = 2.8, p = 0.001; males: ß = 5.1, p < 0.001) was observed. DBP did not show significant relationships with parathormone or either testosterone in males or estradiol in females.

## 4 Discussion

### 4.1 DBP percentiles – age and sex differences

With the current study, we were able to establish age- and sex-specific reference ranges for DBP serum concentrations for healthy children and adolescents. To the best of our knowledge, no other studies have used such a large cohort to publish reference values for DBP levels for healthy children and adolescents. DBP reference values are important for the accurate assessment of vitamin D status, which plays a central role in the development of children and adolescents (3). We estimated age- and sex-specific reference ranges for DBP using 2,067 samples from 1,414 children and adolescents between the ages of 0.25 to 17.98 years. Only a few studies have examined DBP levels in children and adolescents. They found mean values between 330 mg/l and 437 mg/l (30). In reviews by Bouillon et al. and Delanghe et al., DBP serum levels between 200 mg/l and 600 mg/l were reported across all age groups as comparative parameters (3, 15). Variability in DBP serum levels due to the lack of a standardized measurement method makes it challenging to assess DBP’s associations with age, sex, pubertal status, BMI, and other laboratory measurements (3).

### 4.2 DBP’s associations with age, sex, and pubertal status

Our results are in line with most of the published research, which also reported no significant association between DBP levels and age (12). Previous studies also reported higher DBP values in females (31, 32). In our study, DBP levels differed between pubertal stages, especially in male subjects. However, the differences were not substantial, thereby showing, in line with other studies, that DBP is not a good marker of pubertal status (33).

### 4.3 Association between BMI and DBP

Due to the high prevalence of obesity worldwide and in all age groups, we investigated the relationship between BMI and DBP in different stages of life. We found that DBP values decreased as BMI-SDS values increased. Subsequently, the reference cohort subjects showed higher DBP values than the cohort with obesity. The liver is the primary site for the biosynthesis of DBP. Therefore, liver disease, like liver cirrhosis, can sometimes affect protein biosynthesis and result in reduced production of DBP (34). Obesity-related non-alcoholic fatty liver might have similar effects. In the long run, lower levels of DBP could lead to vitamin D deficiency, which is linked to the risk of infections and auto-immune disorders (35). Furthermore, a recent study observed an inverse association of DBP levels with insulin resistance and hyperinsulinemia (36), which can be based on higher BMI levels but also various other reasons such as type 2 diabetes mellitus reported in Moller et al. (37). Higher BMI and lower DBP values might be a risk factor for related pathologies. However, other studies have found no difference in DBP values concerning various BMI groups (12, 38). On the other hand, studies in adults samples have reported positive (10, 39) or negative (4) associations between DBP values and BMI. The size of our cohort may render it more representative than many other studies.

### 4.4 Oral contraceptive use and DBP

We found that oral contraceptives were significantly related to DBP serum levels. This finding lines up with the literature, where a positive association between DBP levels and oral contraceptives has often been reported (2, 6, 31). The DBP serum concentration may increase with estrogen exposure (7, 8, 40). The mechanism behind this effect is not yet fully understood. Oral contraceptives might have positive associations with levels of other binding proteins such as SHBG, trancortin, thyroxine-binding globulin (TBG) (6), and insulin-like growth factor-binding protein 3 (IGFBP-3) (19). These associations suggest an anabolic effect of estrogens on the regulation of hepatic globulin synthesis. Because we also found a small positive association between SHBG and DBP, there might be a common regulatory mechanism. However, oral contraceptives could increase the vitamin D reservoir and thus could prevent vitamin D deficiency. There is evidence that oral contraceptive therapy increases bone density in women with premature ovarian insufficiency (41). Oral contraceptive drugs should also be considered as hormone therapy to prevent diseases such as osteomalacia and osteoporosis. On the other hand, there is evidence that bioavailable vitamin D can be decreased under the use of specific oral contraceptive formulations in adults (42). Given the conflicting research results, it could be hypothesized that females who have used specific formulations of oral contraceptives could have a problem with bioavailable vitamin D, which is compensated for by higher DBP levels. More research is needed to find out.

### 4.5 DBP’s associations with vitamin D metabolites, bone metabolism, acute phase reactants, and protein synthesis

We found that levels of 1,25(OH)_2_-D_3,_ 25(OH)D_3_, and albumin were positively associated with DBP levels, findings that were also previously supported (43, 44). Notably, although DBP increases the total amount of vitamin D metabolites in the bloodstream, it does not appear to affect the amount of the biologically active hormone that enters cells and tissues (45). It is important to note that different polymorphisms of DBP also have different affinities for vitamin D metabolites. The prevalence of the isoforms differs by ethnic origin (46), which is why the biological relevance of DBP-bound vitamin D metabolites versus the DBP-unbound or free fraction of vitamin D has not been definitively established (3, 47). Bone parameters’ associations with age, sex, pubertal status, and BMI were recently described by Geserick et al. (48). The associations between measured parameters and DBP found in our study were very small but still significant. Since bioavailable vitamin D induces the formation of osteocalcin (49), the inverse relationship between osteocalcin and DBP could indicate that DBP is part of a negative-feedback-based regulation of the total amount of vitamin D despite the finding that this effect was small. A positive association between DBP levels and acute phase reactants such as CRP or interleukin-6 has already been reported in various studies (5, 39, 50). It was speculated that higher CRP levels in people with obesity may be related to higher DBP serum levels, but our findings did not support this idea. Rather, our results support the hypothesis that DBP is part of the acute phase response in which liver protein synthesis of acute phase proteins is upregulated by proinflammatory cytokines (50, 51). However, Madden et al. reported lower DBP levels during severe infections (30). The association between CRP and DBP was positively significant, even at slightly elevated CRP levels (<20 mg/l), suggesting DBP may indicate minor inflammatory processes. The relationship between DBP and inflammatory cytokines may also depend on the severity and duration of inflammation, as DPB plays a major role in inflammation and immune cell modulation as an acute-phase reactant itself.

### 4.6 Strength and limitations

The main strength of the current study is the large number of subjects. Furthermore, all laboratory analyses were performed in only one facility. The subjects were mostly Caucasian and lived near or in the city of Leipzig. The results were based on the social distribution of the city of Leipzig, and therefore, the estimated reference ranges may be valid only for comparable populations. To include regional and social variety, other studies should be considered. It is essential to consider the limited amount of data available for children under 4.5 years of age when applying reference values from 0.25 years to 17.98 for the reference cohort.

## 5 Conclusion

DBP is essential for evaluating vitamin D status. DBP levels decrease with higher BMI and in late male puberty but increase with higher CRP levels and oral contraceptive use. These factors should be considered when interpreting DBP levels to ensure accurate vitamin D assessments. However, the percentiles identified in this study suggest that DBP serum concentrations are a relatively stable parameter across age with small variation. This study is important to determine the norms of DBP in controls for later comparisons with different pathologies.

## Data Availability

The raw data supporting the conclusions of this article will be made available by the authors, without undue reservation.

## Supplementary material

Supplementary Figure 1 Correlation plot showing the correlations between bone parameters, C-reactive protein, and hormones. Pearson correlation coefficients are distinguished by color (red = negative correlation, blue = positive correlation). Represented by the dendrograms, hierarchical clustering groups together parameters that are strongly correlated with each other. Stronger relationships are indicated by bordered groups and more intense colors.
