# Supplementary figures and images for: Effect of sex, pubertal stage, body mass index, oral contraceptive use, and C-reactive protein on vitamin D binding protein reference values
Source: Front Endocrinol (Lausanne). 2025 Feb 18;16:1470513. doi: 10.3389/fendo.2025.1470513 (PMC11876044; doi:10.3389/fendo.2025.1470513)

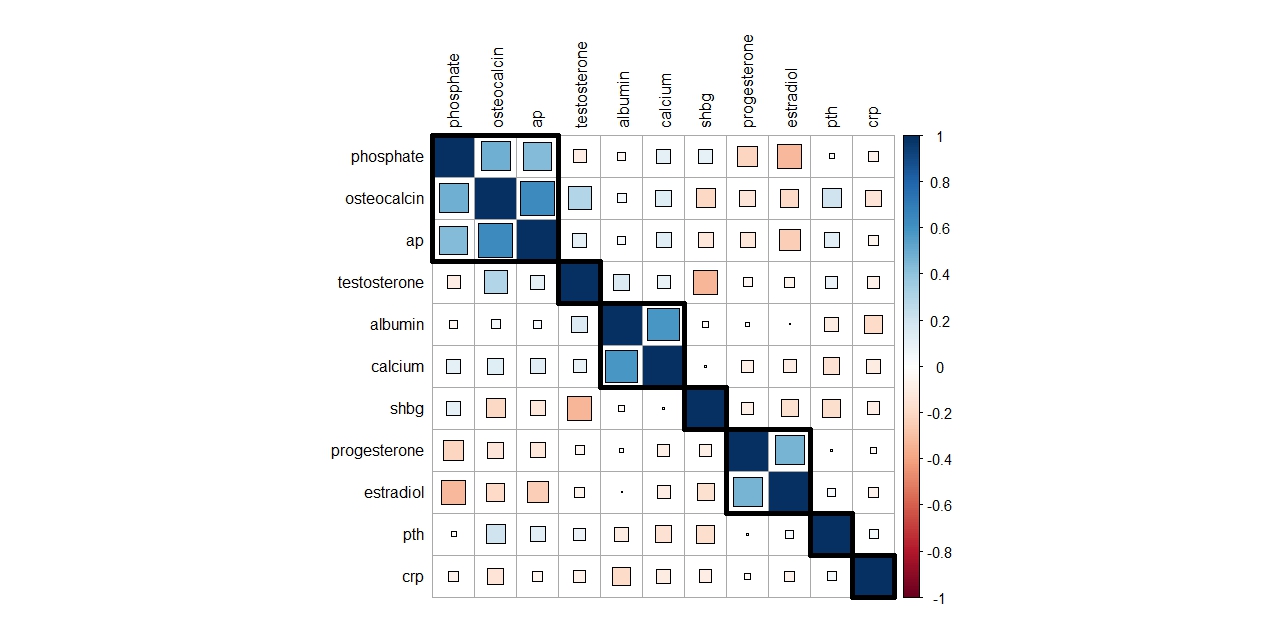

Supplement: Supplementary Figure 1 — Correlation plot showing the correlations between bone parameters, C-reactive protein, and hormones. Pearson correlation coefficients are distinguished by color (red = negative correlation, blue = positive correlation). Represented by the dendrograms, hierarchical clustering groups together parameters that are strongly correlated with each other. Stronger relationships are indicated by bordered groups and more intense colors. [file Image1.jpeg]
