# Supplementary material for: Effect of sex, pubertal stage, body mass index, oral contraceptive use, and C-reactive protein on vitamin D binding protein reference values
Source: Front Endocrinol (Lausanne). 2025 Feb 18;16:1470513. doi: 10.3389/fendo.2025.1470513 (PMC11876044; doi:10.3389/fendo.2025.1470513)
Supplement: Supplementary file 2 [file Table1.docx]

Suppl. Table 4: Mean DBP levels stratified by sex and Tanner stages. Note: p-values. p = significance codes: < 0.001 “***”, < 0.01 “**”, < 0.05 “*”, Abbreviations: DBP: Vitamin D binding protein

|  | **mean DBP (mg/l)** | |  |
| --- | --- | --- | --- |
|  | **females** | **males** | **p-value** |
| Tanner 1 | 355 | 350 | 0.143 |
| Tanner 2 | 355 | 356 | 0.773 |
| Tanner 3 | 357 | 337 | < 0.05 * |
| Tanner 4 | 362 | 331 | < 0.001 *** |
| Tanner 5 | 357 | 332 | < 0.01 ** |

Suppl. Table 5: Reference cohort compared with obese cohort with no subjects taking oral contraceptives. Note: estimate = ß, p-values. p = significance codes: < 0.001 “***”, < 0.01 “**”, < 0.05 “*”, DBP in mg/l, “reference” indicates the reference value. Abbreviations: DBP: Vitamin D binding protein, n: number of samples, na: not available

| **reference females** | **Tanner**  **stage** | **n** | **mean DBP (mg/l)** | **estimate** | **p** |  | **obese females** | | **Tanner stage** | | **n** | | **mean DBP (mg/l)** | | **estimate** | **p** |
| --- | --- | --- | --- | --- | --- | --- | --- | --- | --- | --- | --- | --- | --- | --- | --- | --- |
|  | 1 | 384 | 356 |  | reference |  | |  | 1 | 33 | | 333 | |  | | reference |
|  | 2 | 158 | 354 | -2.02 | 0.65 |  | |  | 2 | 23 | | 363 | | 29.60 | | 0.03* |
|  | 3 | 124 | 360 | 3.78 | 0.44 |  | |  | 3 | 20 | | 347 | | 13.50 | | 0.34 |
|  | 4 | 128 | 364 | 7.64 | 0.12 |  | |  | 4 | 21 | | 347 | | 1.41 | | 0.92 |
|  | 5 | 121 | 360 | 3.43 | 0.49 |  | |  | 5 | 43 | | 354 | | 20.50 | | 0.11 |
|  | na | 71 |  |  |  |  | |  | na | 18 | |  | |  | |  |
| **reference males** |  |  |  |  |  |  | | **obese males** |  |  | |  | |  | |  |
|  | 1 | 458 | 352 |  | reference |  | |  | 1 | 46 | | 330 | |  | | reference |
|  | 2 | 140 | 360 | 7.12 | 0.10 |  | |  | 2 | 28 | | 333 | | 2.91 | | 0.82 |
|  | 3 | 38 | 346 | -6.40 | 0.39 |  | |  | 3 | 17 | | 319 | | -11.00 | | 0.46 |
|  | 4 | 60 | 336 | -16.50 | 0.01* |  | |  | 4 | 12 | | 310 | | -19.30 | | 0.25 |
|  | 5 | 37 | 341 | -11.20 | 0.16 |  | |  | 5 | 12 | | 310 | | -19.80 | | 0.23 |
|  | na | 348 |  |  |  |  | |  | na | 44 | |  | |  | |  |
